# Achiral Molecular Recognition of Substituted Aniline Position Isomers by Crown Ether Type Chiral Stationary Phase

**DOI:** 10.3390/molecules26020493

**Published:** 2021-01-18

**Authors:** Atsushi Ohnishi, Tohru Shibata, Tatsuya Imase, Satoshi Shinkura, Kanji Nagai

**Affiliations:** 1 R&D Center, Analytical Tools BU, CPI Company, Daicel Corporation, Myoko, Niigata 944-8550, Japan; kn_nagai@jp.daicel.com; 2 Graduate School of Natural Science and Technology, Kanazawa University, Kakuma, Kanazawa 920-1192, Japan; 3 Global R&D Group, CPI Company, Daicel Corporation, Himeji, Hyogo 671-1283, Japan; tr_shibata@jp.daicel.com (T.S.); sa_shinkura@jp.daicel.com (S.S.); 4 Analytical Solution, Corporate Research Center, Innovation and Business Development Headquarters, Daicel Corporation, Himeji, Hyogo 671-1283, Japan; tt_imase@jp.daicel.com

**Keywords:** CROWNPAK CR-I, chromatography, crown ether, aniline, positional isomer, quantum chemical calculation, three-point binding model, chiral separation

## Abstract

To understand the selectivity of the crown ether type chiral stationary phase (CSP), the retention selectivity for aniline and the positional isomers of substituted anilines were studied. In various substituted isomers, except nitroaniline, a remarkable decrease of retention due to steric hindrance was observed for the 2-substituted isomer. To determine the detailed molecular recognition mechanism, quantum chemical calculations were performed for the aggregates between the crown ether and the anilines. The results suggested that the 20-Crown-6, which includes a phenyl-substituted 1,1′-binaphthyl moiety, interacts with alkyl and aryl amines in an unconventional form different from the proposed one for 18-Crown-6.

## 1. Introduction

In recent years, many kinds of chromatography phases have become available, and this has aroused interest in the orthogonality among the phases. We expected that chiral stationary phases would have distinct orthogonality with many achiral phases stemming from the mechanism of molecular recognition [1,2,3]. Originally, chiral stationary phases were developed for the purpose of enantiomeric separation and were primarily used in the optical purity measurement of an enantiomeric mixture [4]. The idea of applying these chiral stationary phases to achiral separation is not particularly novel, but it is not so popularly applied in reality.

The most important reason for the underdevelopment appears to be that it is not easy to predict the separation selectivity of a chiral stationary phase (CSP) because the understanding of the separation mechanism is still insufficient. In general, chiral stationary phases recognize the molecular shape and many achiral phase physicochemical properties, such as the hydrophilicity/hydrophobicity balance by octadecylsilyl (ODS) phase and polarity by silica gel. Therefore, a CSP was expected to have good potential to separate molecules with similar physicochemical properties, such as isomers, and such an expectation is now being proved.

For the potential of achiral separation by CSPs to be practically utilized as described above, elucidation of its selectivity or separation mechanism would contribute significantly. We previously reported the elucidation of the recognition mechanism by the chromatography of non-chiral compounds, such as aromatic positional isomers and computational simulation, focusing on polysaccharide derivative-based CSPs. We demonstrated the unique molecular shape recognition of the polysaccharide derivative-based CSPs, which are different from an ODS phase [5].

**Scheme 1:**
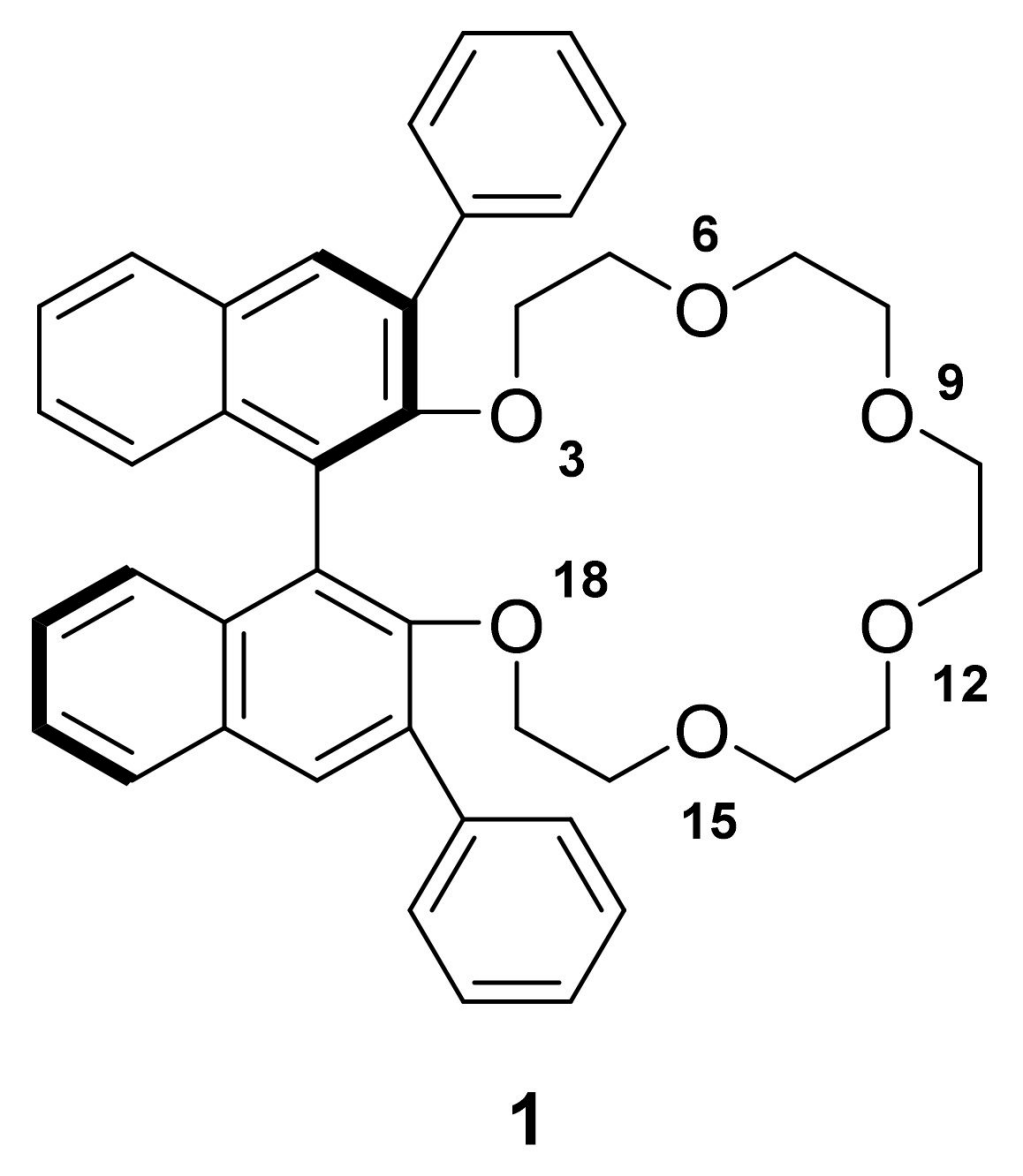
Ligand of CROWNPAK CR-I (-), *(R)*-(−)-(3,3′-diphenyl-1,1′-binaphtyl)20-crown-6 (**1**). (Tethering is omitted.).

In this report, we show the selectivity of a crown ether type CSP, CROWNPAK CR-I (CR-I, Scheme 1), for isomeric substituted anilines and the related compounds. This type of CSP is known to have a higher chiral recognition ability for enantiomers having a primary amino group and to be particularly useful for the enantioseparation of natural and non-natural amino acids [6,7,8,9]. The chiral recognition of chiral crown ethers was developed by Cram et al. [10]. The first commercially available CSP, CROWNPAK CR, was developed by Shinbo et al. and was called a coating type [11], and then other crown ether type CSPs were reported [12,13,14].

In this CROWNPAK CR (hereafter abbreviated as CR), a hydrophobic crown ether as a chiral selector was dissolved into the lipophilic layer of a reversed-phase silica gel to suppress elution into an eluent. However, strict restrictions are imposed on the eluent for the CR phase, that is, only aqueous methanol of a concentration lower than 15% (*v/v*) can be used and even acetonitrile (ACN), which is used exclusively in the reverse-phase high-performance liquid chromatography (HPLC) on an ODS column, cannot be used. For this reason, it often occurs that highly hydrophobic compounds take a long time for analysis or even not be eluted.

On the other hand, immobilized CROWNPAK CR-I is free from such restrictions, and both a water-based eluent and an ACN-based or a hexane-based eluent can be applied. This has made the optimization of a targeted separation much easier and, for example, made the analysis time of a highly hydrophobic compound reasonable.

In this study, we took up this CR-I phase as the control of the elution with the eluent polarity is particularly important in achiral separation. Therefore, we could discuss the results in terms of the analyte structure and the relation to the eluent property. In addition to the chromatographic study, we attempted simulation by quantum chemical calculation of what is occurring in the chromatographic molecular recognition. As a result, we proposed a new interaction model for this type of crown ether, which is different from the three-point binding mechanism that has been widely accepted as the molecular recognition model for 18-crown-6 as proposed by Cram et al. [15].

## 2. Results and Discussions

### 2.1. Separation of Aniline and Substituted Aniline Isomers on CROWNPAK CR-I

#### 2.1.1. Separation of Aniline and Substituted Anilines

**Scheme 2:**
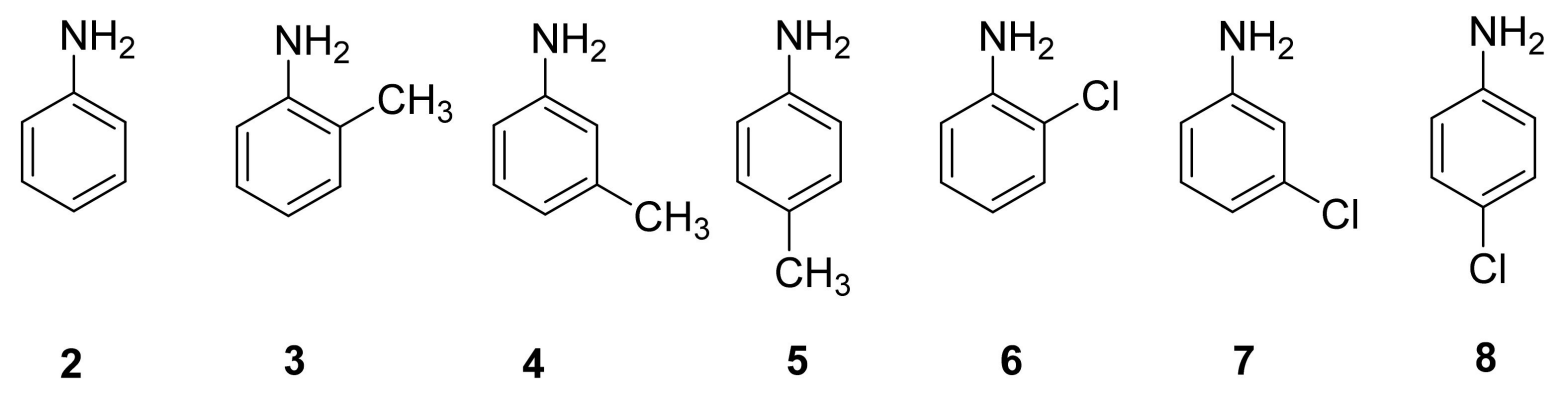
Structure of the analytes appearing in Table 1.

**Figure 1:**
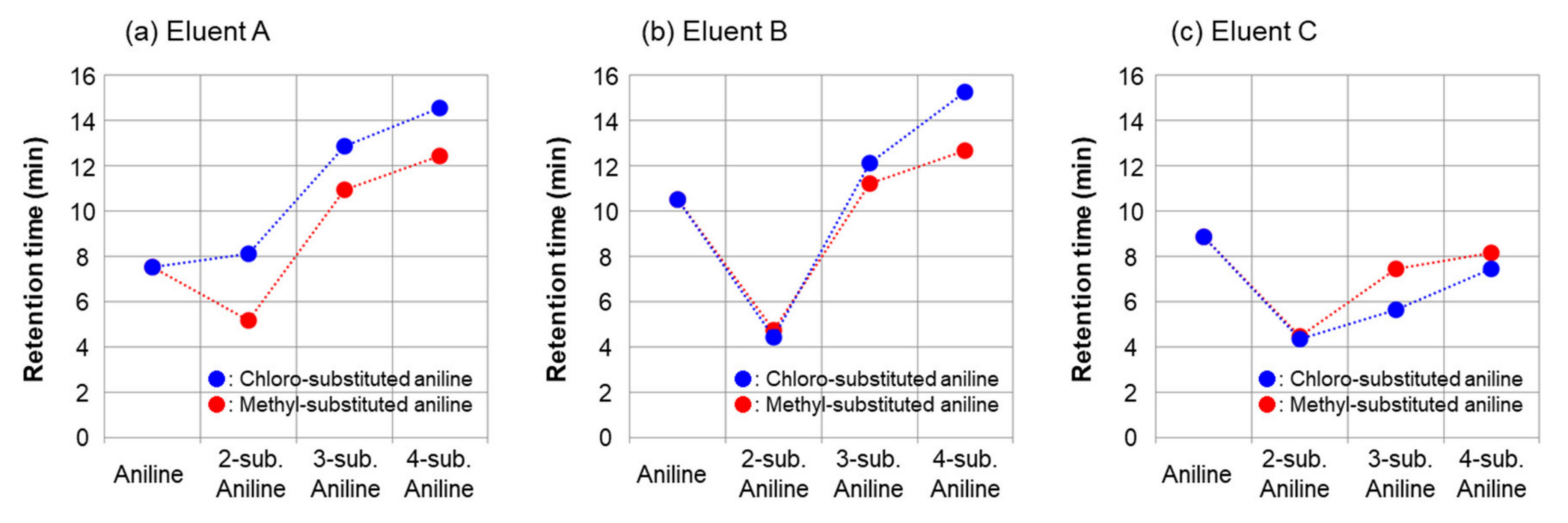
Plots of the retention times of aniline (**2**), methylaniline isomers (**3**–**5**), and chloroaniline isomers (**6**–**8**) evaluated using a CROWNPAK CR-I(-) column. The eluents are (**a**) eluent A, (**b**) eluent B, and (**c**) eluent C. The structures of the analytes are shown in Scheme 2.

**Figure 2:**
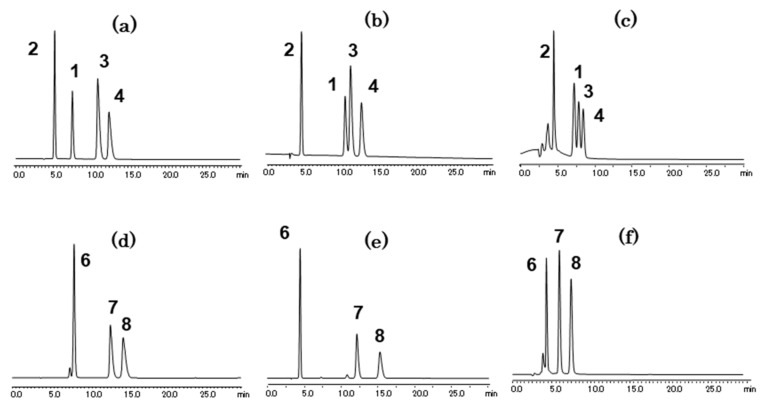
Reverse-phase high-performance liquid chromatography (HPLC) chromatograms of aniline (**2**), methylaniline isomers (**3**–**5**), and chloroaniline isomers (**6**–**8**) evaluated using a CROWNPAK CR-I(-) column. The eluents are (**a**,**d**) eluent A, (**b**,**e**) eluent B, and (**c**,**f**) eluent C. The structures of the analytes are shown in Scheme 2.

**Scheme 3:**
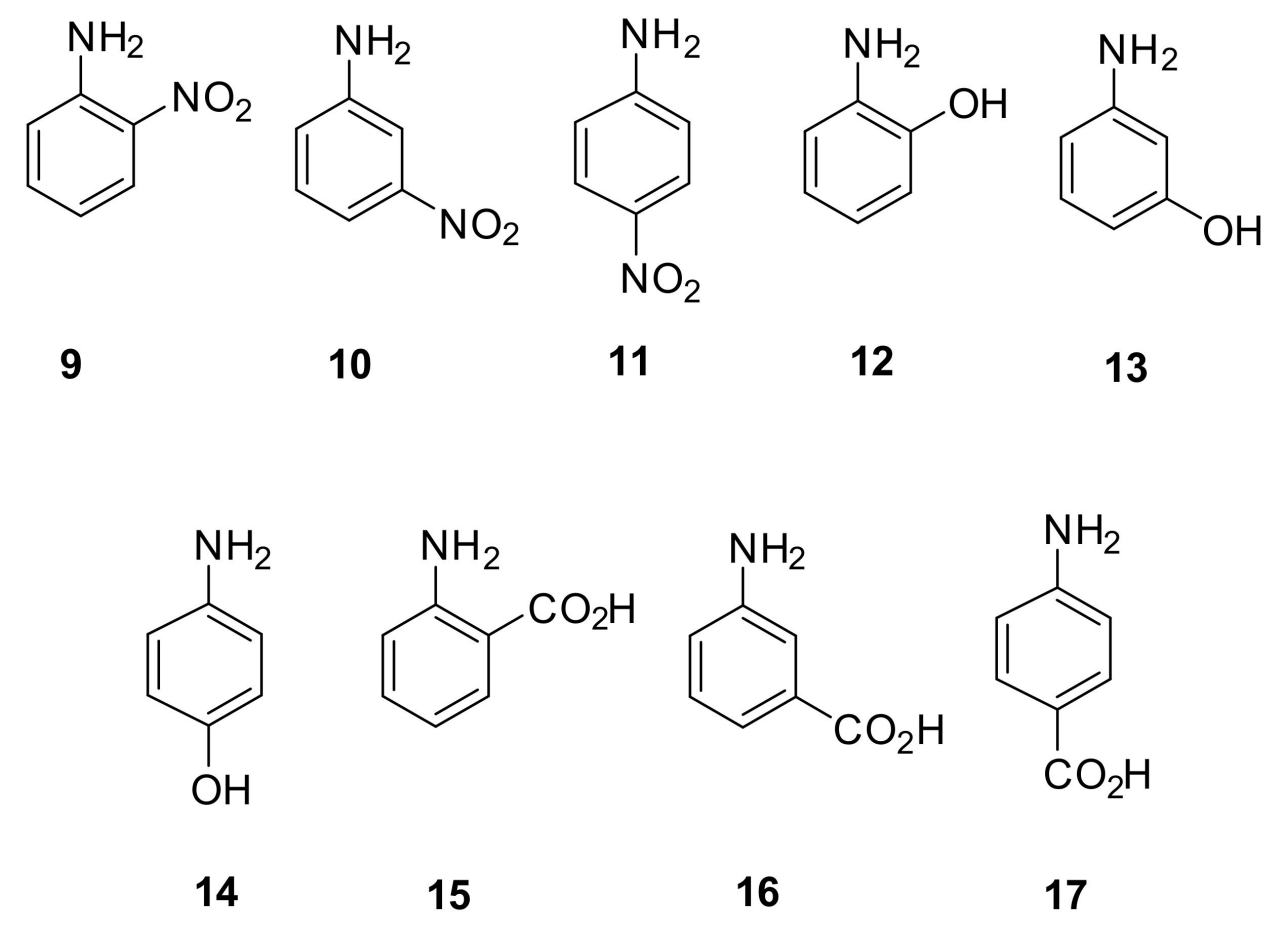
Structure of the analytes appearing in Table 2.

**Figure 3:**
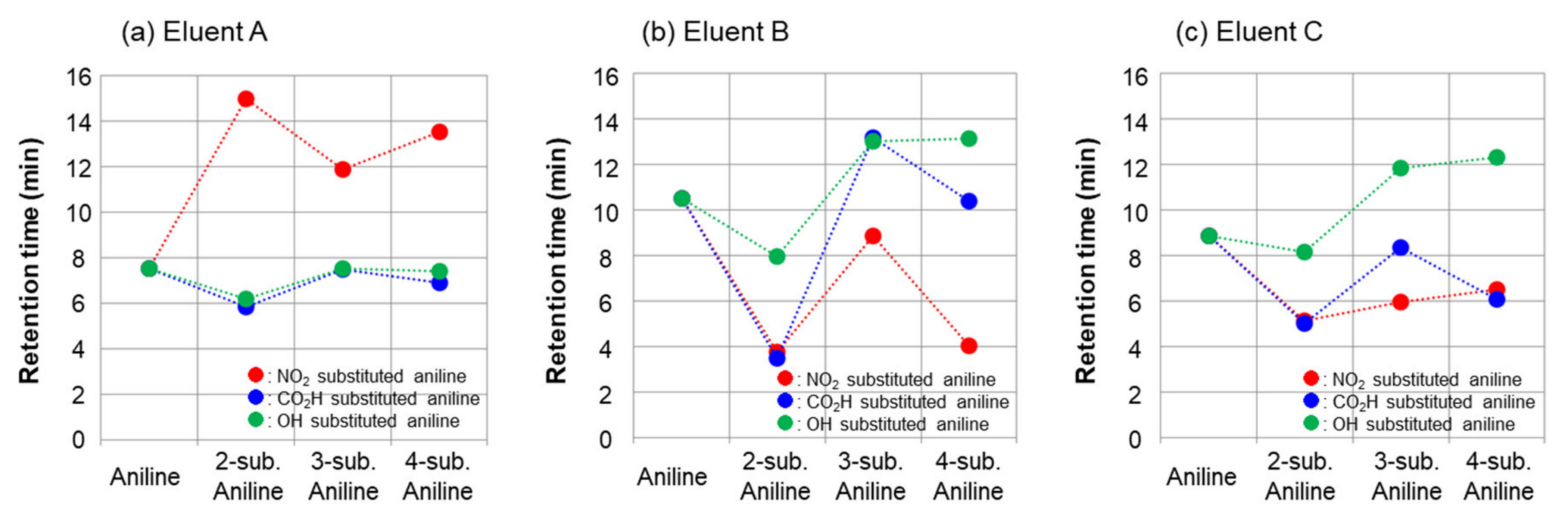
Plots of the retention times of aniline (**2**), nitroaniline isomers (**9**–**11**), hydroxyaniline isomers (**12**–**14**), and carboxyaniline isomers (**15**–**17**) evaluated using a CROWNPAK CR-I(-) column. The eluents are (**a**) eluent A, (**b**) eluent B, and (**c**) eluent C. The structures of the analytes are shown in Scheme 3.

Table 1 and Table 2 show the retention times of unsubstituted aniline, non-polar group-substituted anilines, and polar group-substituted anilines (Scheme 2 and Scheme 3) under three kinds of eluent (A, B, and C). The composition of each eluent is given below and other chromatographic conditions are in Section 3. Material and Methods. The line graphs and chromatograms (Figure 1, Figure 2 and Figure 3) with the retention time on the vertical axis and the analytes on the horizontal axis are also shown for aiding visual understanding.

Eluent A: 20 mM HClO_4_ in 70:30 (*v*/*v*) mixture of H_2_O and ACN

Eluent B: 20 mM HClO_4_ in 10:90 (*v*/*v*) mixture of H_2_O and ACN

Eluent C: 100/100/4/1 (*v*/*v*/*v*/*v*) mixture of n-hexane, ethanol, water, and trifluoroacetic acid

These eluents, A, B, and C are the recommended ones in the instruction manual published by Daicel. Perchloric acid is one of the acids resulting in good retention and separation, and this will be discussed elsewhere [16].

Retention behavior of these aniline and substituted anilines on CR-I(-) may be outlined with the following two features. One is concerned with the retention of isomeric forms. In each case, except for nitroanilines (vide infra), 2-substituted aniline isomer is retained considerably less than 3- and 4-substituted aniline isomers, which are separated moderately. This characteristic recognition pattern was also seen with a mixture of water/methanol (70:30 *v/v*) containing 20 mM HClO_4_ as the eluent. This strongly suggests the steric hindrance by the substituent at 2-position against the interaction with the crown ether and features the selectivity of the CR-I phase for substituted anilines.

This selection rule is no more apparent for benzylamines bearing a methyl substituent at the 2-position on its benzene ring (vide Section 2.1.2). Another feature is concerned with the effect of substitution in relation to the eluent polarity. Broadly speaking, regarding the retention of 3- and 4-substituted anilines, a hydrophobic substituent (i.e., methyl and chlorine) makes the retention stronger than bare aniline with an aqueous eluent, A, and a hydrophilic one (i.e., hydroxyl and carboxy) with an organic solvent-based eluent, B and C. The sum of the above effects, the steric hindrance, and the polarity balance between the analyte and eluent broadly determine the actual retention. The polarity balance observed suggests the amphiphilic property of the retention interaction.

**Figure 4:**
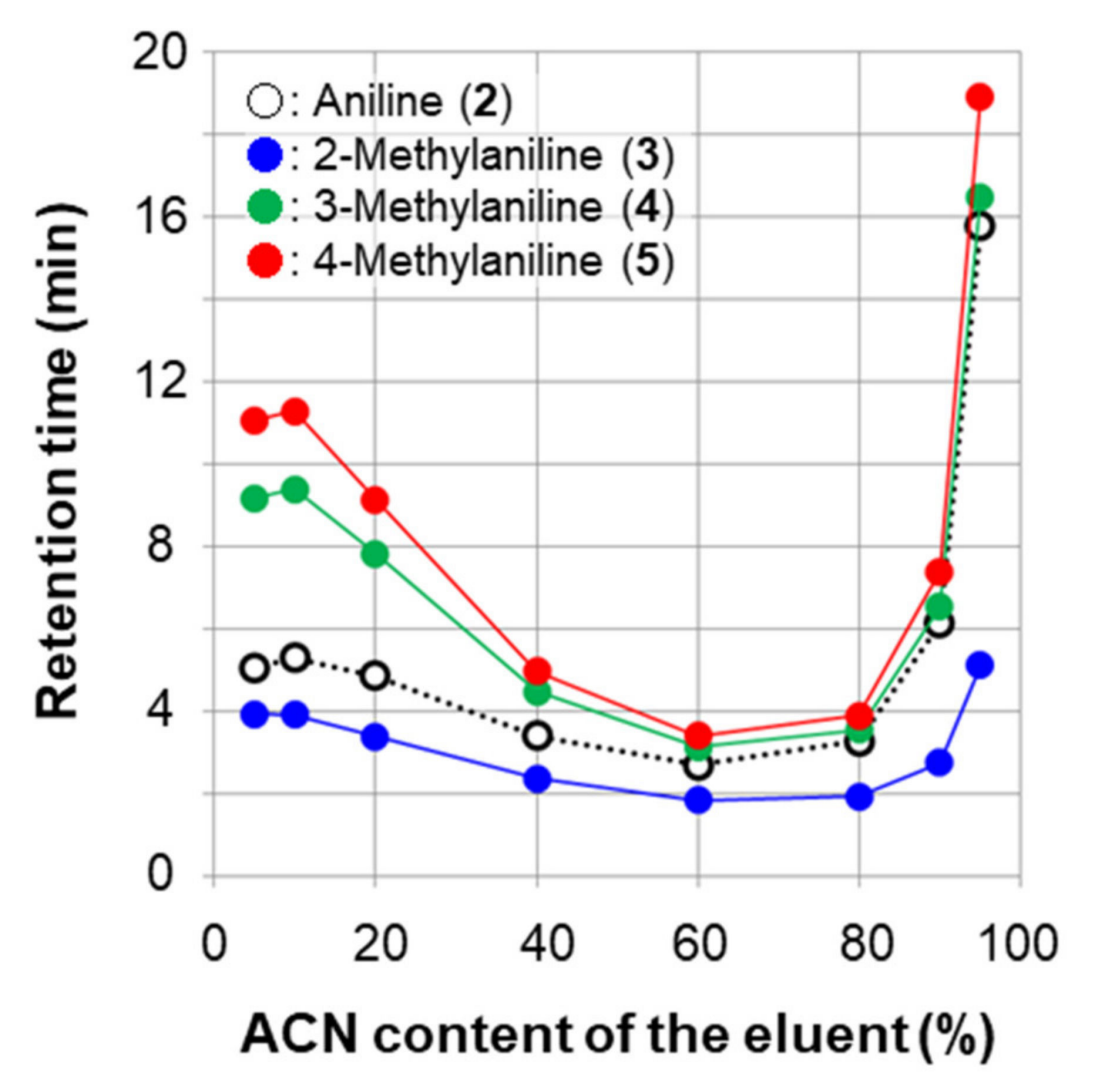
Effect of the volume fraction of acetonitrile (ACN) in the eluent on the retention time of aniline (**2**) and methylaniline isomers (**3**–**5**). Column: CROWNPAK CR-I(-). Eluent: ca 17.1 mM HClO_4_ in a mixture of H_2_O and ACN at a flow rate of 0.43 mL/min.

Therefore, the dependence of the analyte retention on the eluent composition with a constant concentration of perchloric acid was investigated. Figure 4 is the plot of the retention time of the analytes versus the ACN/water composition (*v/v*), and the plots exhibit U-shaped retention curves as shown by Konya and Fukusaki et al. for amino acids with eluents containing trifluoroacetic acid [7]. While the usefulness of ACN-rich eluent B was previously exhibited by Konya and Fukusaki [8], we emphasize again the merit of CR-I with which a considerably wider range of polar and nonpolar eluents can be adopted.

However, the retention of nitroaniline isomers was apparently out of the line of the above discussion. This appears to be due to the extremely weak basicity. The pKa values of nitroaniline isomers were reported to be −0.26, 2.466, and 1.0 for 2-, 3-, and 4-nitroanilines, respectively [17]. The pH of aqueous 20 mM perchloric acid was calculated to be 1.70 (in pure water), and the protonation of at least 2- and 4-nitroanilines must occur only partially in such an eluent. Therefore, the retention at least of 2- and 4-nitroanilines appears to be of the neutral (unprotonated) species.

The result supporting this understanding is the retention of substituted anilines with a neutral buffered eluent at pH 7.6. With this eluent, the retention of nitroaniline isomers was not much altered, while the retention pattern among isomers of other substituted anilines was drastically changed [16]. Thus, we rationalized that the characteristic retention pattern, particularly the largest retention of the 2-isomer among isomeric nitroanilines with eluent A, is not the result of ammonium ion interactions with the crown moiety but the result of neutral molecular adsorption presumably by hydrophobic interaction.

**Figure 5:**
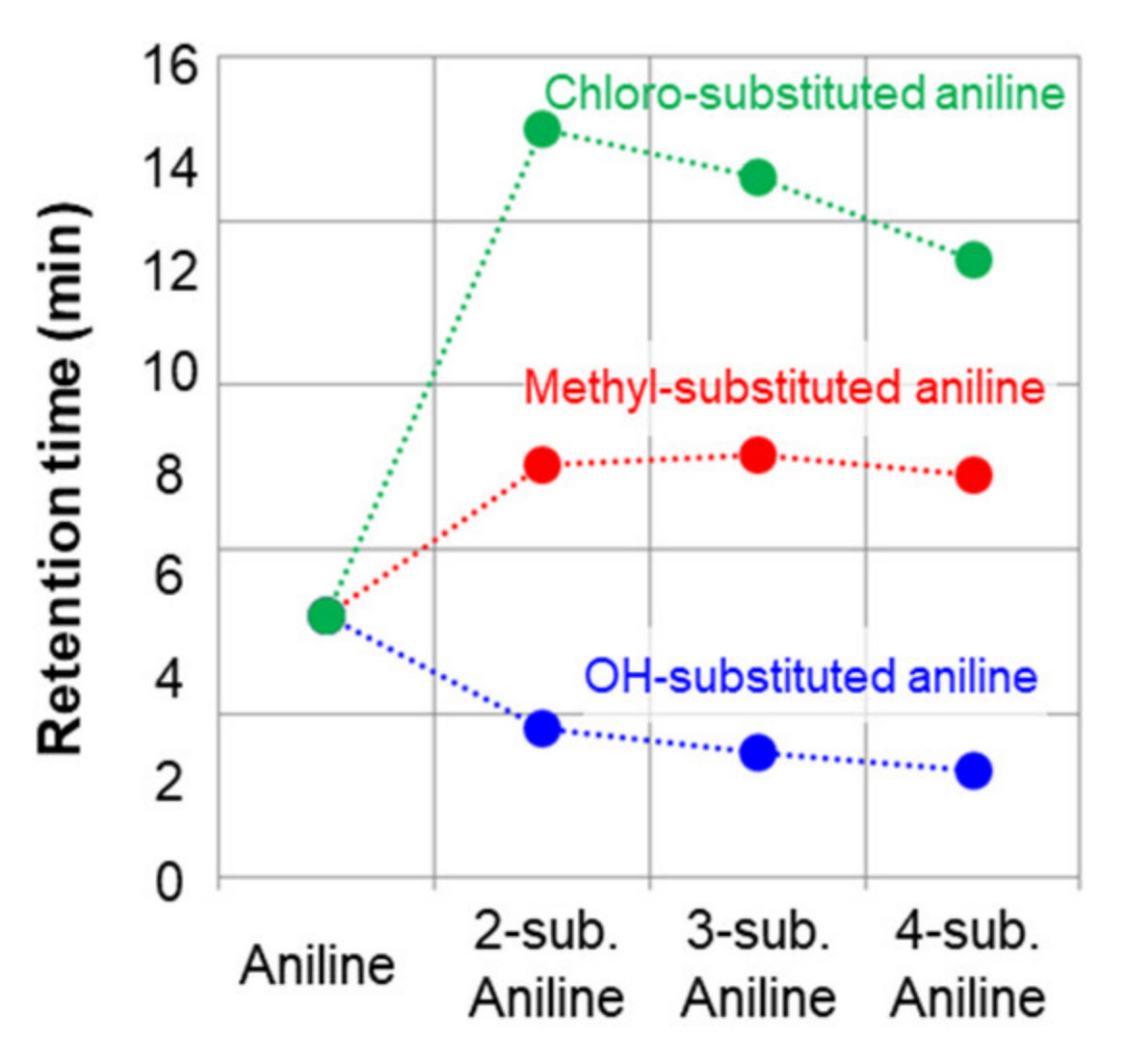
Plots of the retention times of aniline (**2**) and its methyl-, (**3**–**5**), chloro- (**6**–**8**), and hydroxy-substituted (**12**–**14**) isomers evaluated using an ODS column. The eluent composition was 20 mM phosphoric acid and 30 mM triethylamine in a 70:30 (*v/v*) mixture of H_2_O and ACN.

In this connection, the retention of substituted aniline isomers on a common ODS phase was studied under the analytical conditions given in Section 3. Materials and Methods. Table 3 shows the retention times of unsubstituted aniline (**2**) and certain substituted anilines (**3**–**8**), (**12**–**14**) under 20 mM neutral phosphate buffer (pH 7.6). Figure 5 shows the retention pattern of substituted aniline isomers and 2-substituted anilines, which exhibited a higher or roughly the same retention in comparison with other isomers. The recognition of isomerism was generally moderate in contrast with the retention selectivity of CR-I.

#### 2.1.2. Separation of Benzylamine and Substituted Benzylamines

**Scheme 4:**
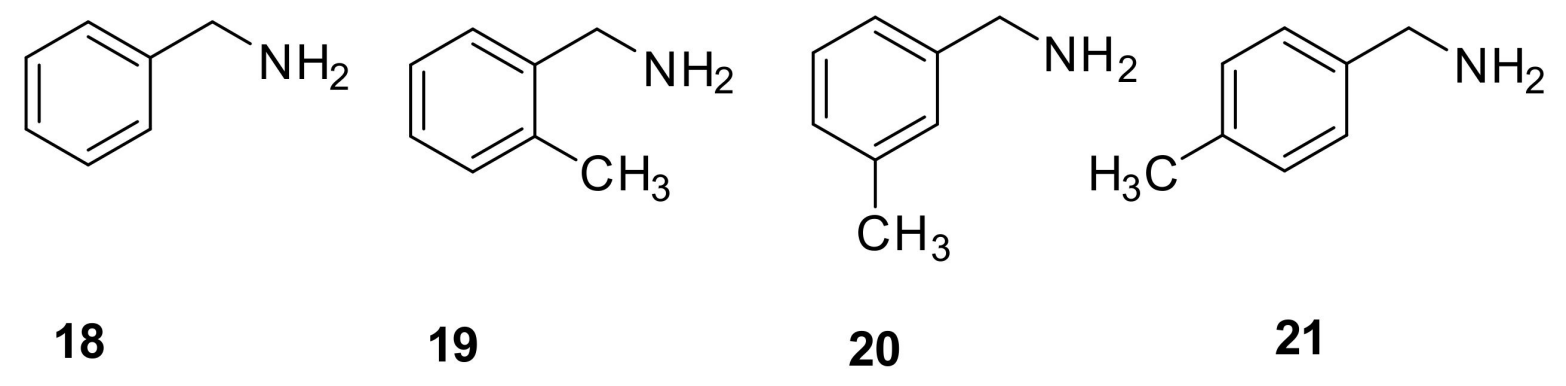
Structure of the analytes appearing in Table 4.

**Figure 6:**
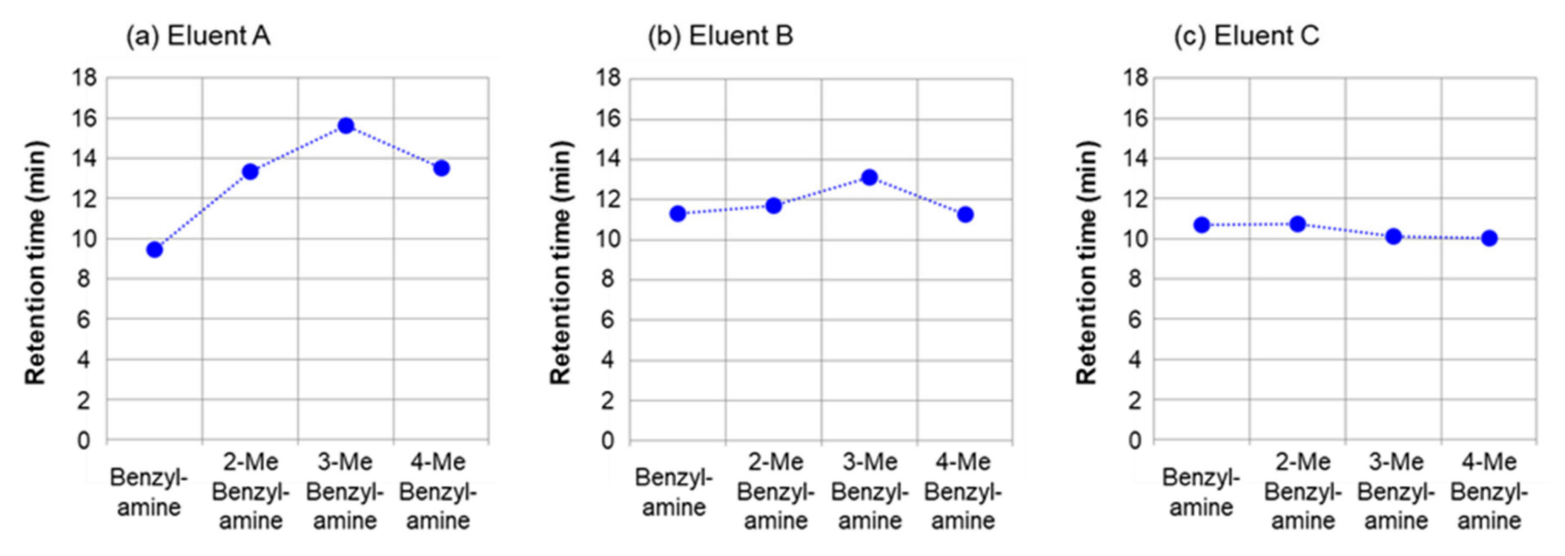
Plots of the retention times of benzylamine (**18**) and its methyl-substituted isomers (**19**–**21**) evaluated using a CROWNPAK CR-I(-) column. The eluents are (**a**) eluent A, (**b**) eluent B, and (**c**) eluent C.

**Figure 7:**
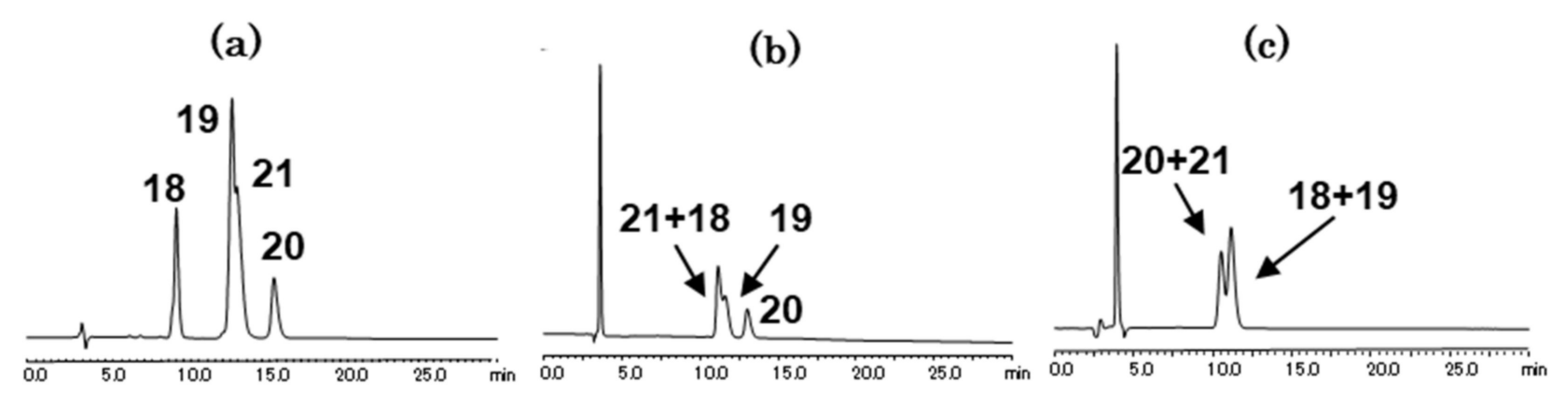
HPLC chromatograms of benzylamine (**18**) and substituted benzylamine isomers (**19**–**21**) evaluated by using a CROWNPAK CR-I(-) column. The eluents are (**a**) eluent A, (**b**) eluent B, and (**c**) eluent C. The structures of the analytes are shown in Scheme 4.

Chromatographic evaluations of benzylamine (**18**) and methyl-substituted benzylamines (19–21) on CROWNPAK CR-I(-) were performed under the same HPLC conditions as the aniline analysis (Scheme 4, Table 4, Figure 6 and Figure 7). Table 4 shows the retention times in Scheme 4. No significant decrease in the retention caused by the substituent at the 2-position was observed. The insertion of one methylene group—that is, a longer distance between the amino group and methyl substituent on benzene ring—presumably relieved the steric hindrance effect observed in substituted anilines. Methyl-substituted benzylamine, which is expected to be more hydrophobic than unsubstituted benzylamine, was retained more than the bare benzylamine with eluent A, slightly more or even less with eluent B and C—that is, the relationship with eluent polarity as was observed in the case of anilines.

### 2.2. Examination of Retention Behavior by Quantum Chemical Calculation

Section 2.1 described that the retention behaviors of aniline, substituted aniline isomers, and benzylamines on CROWNPAK CR-I (-) can be explained based on two points—the steric hindrance and relative polarity of the eluent and analytes. In this section, we consider the relationship among their elution behaviors and the stabilization energies and interaction model obtained by a quantum chemical calculation with the solvent effect of water.

**Scheme 5:**
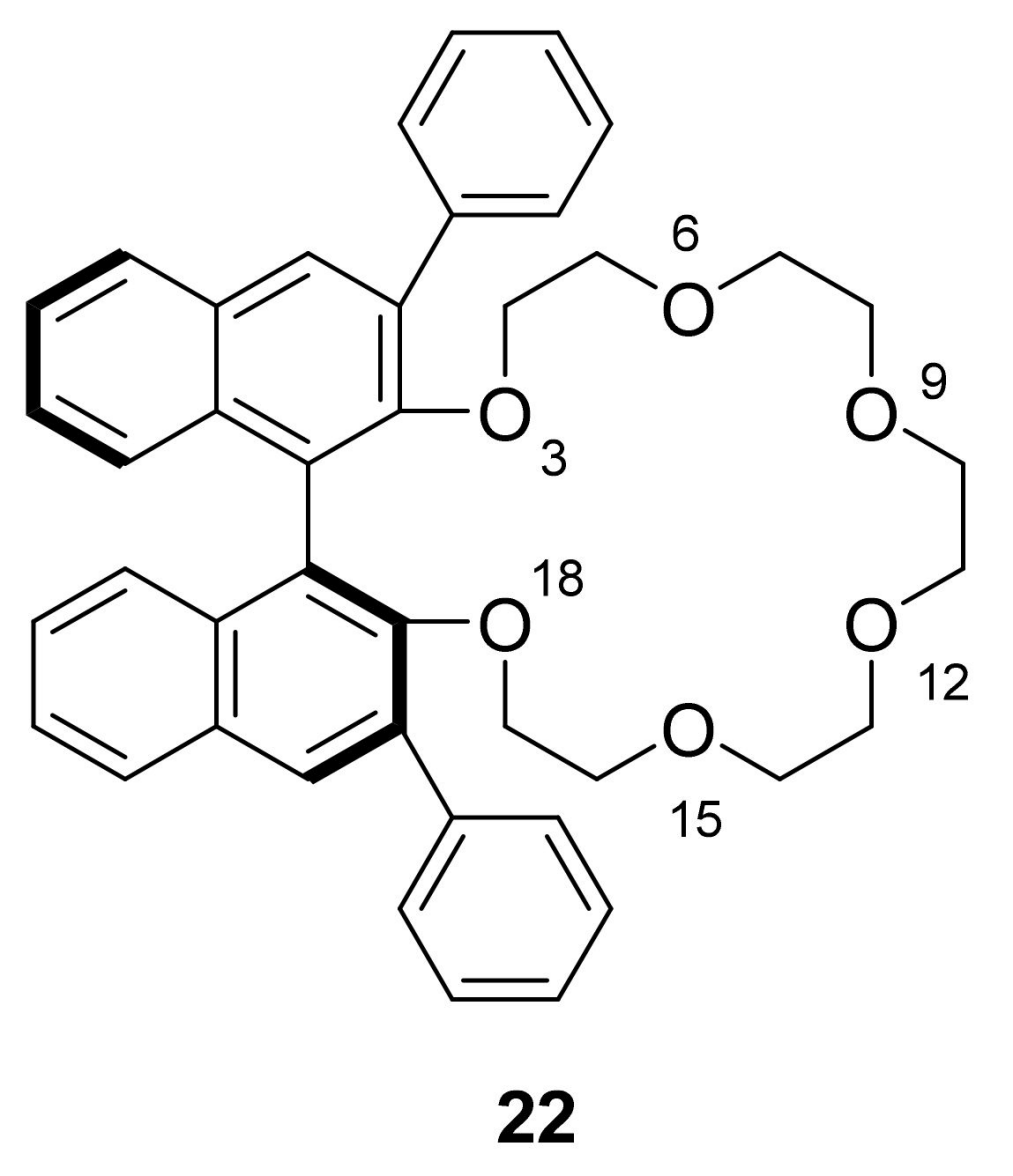
Ligand structure of CR-I (+), *(S)*-(+)-(3,3-diphenyl-1,1′-binaphtyl)-20-crown-6 (**22**). (Tethering is omitted.).

While a CR-I(-) column was used in the above-mentioned chromatographic study, in the following in silico study, the crown ether **22**, the selector of a CROWNPAK CR-I(+) column in Scheme 5, was taken as the model. This is because a CR-I(+) column is more often used as the elution order, D-amino acid first is usually more convenient in the chiral analysis of amino acids. Needless to say, CR-I(+) should give the same selectivity in achiral separation.

First, the calculation for the optimized structure of **22** was performed using the force field calculation by molecular mechanics-3 (MM-3) [18]. Ten types of crown ether structures were extracted as metastable structures with the largest stabilization energies. The quantum chemical calculations (described in Section 3. Materials and Methods) were applied to these 10 types of structures as an initial structure.

Consequently, the most stable structure among them was determined. An ammonium ion was placed at arbitrary positions on each molecule, and the stable structure of the aggregate was obtained by performing the quantum chemical calculations at the same level of assuming in water (dielectric constant ε = 78.39). In some cases, we confirmed that, even if the position of an ammonium ion in the initial structure of the aggregate was arbitrarily changed, the aggregate structure converged to the same stable one after optimization.

#### Quantum Chemical Calculation of Aniline, Methyl, and Chloroanilines

**Figure 8:**
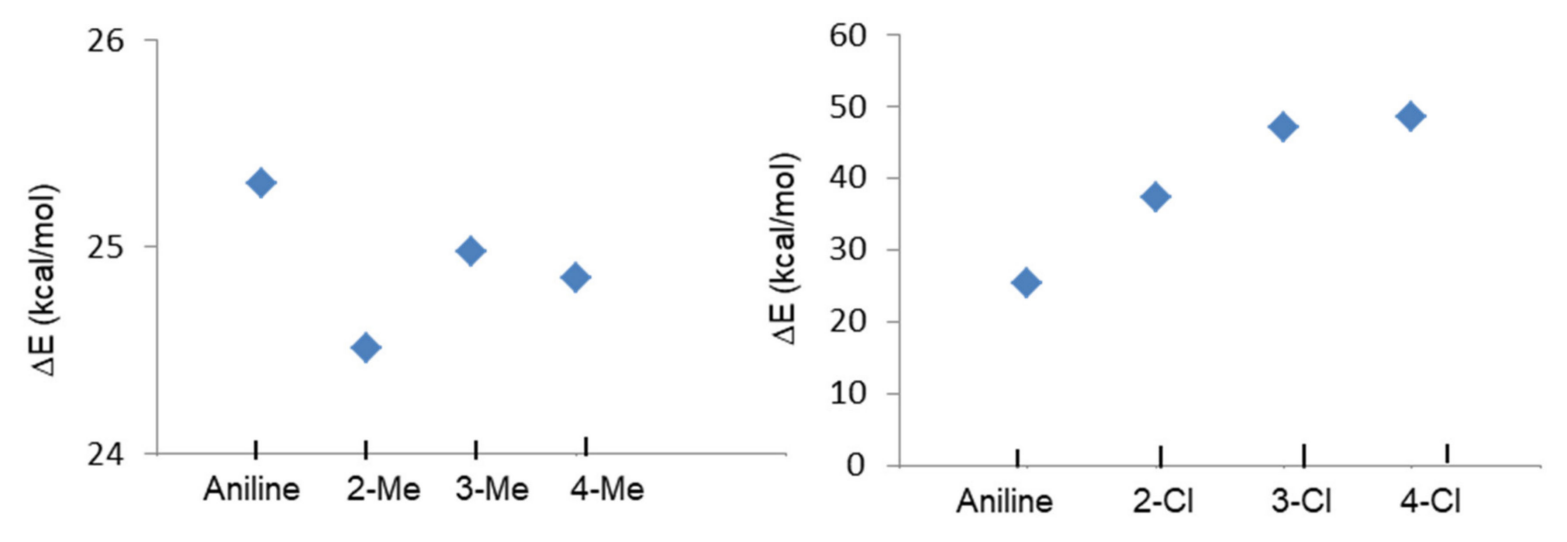
Stabilization energy between anilines and CR-I(+) (**22**), aniline and methylanilines (**left**), and aniline and chloroanilines (**right**).

The aggregate structure between CR-I(+) and substituted anilines and the stabilization energy was determined by quantum chemical calculation. The calculated stabilization energies well supported the decreased retention of substituted anilines at the 2-position, which can be characterized as the feature of CR-I selectivity as shown in 1-1 (see Figure 1 and Figure 3). Surprisingly, this form of aggregate was significantly different from the three-point binding form (tripods) proposed by Cram et al. [15], which will be discussed in detail in the next section. Table 5 shows the stabilization energies of aniline and aniline derivatives, and Figure 8 shows each stabilization energy on the vertical axis and the analytes on the horizontal axis.

As shown in Figure 1a and Figure 8, a good agreement was found between the stabilization energy and the retention of substituted isomers for both methylanilines and chloroanilines. Among them, the lowest stabilization energy (unstable) and short retention time of the anilines substituted at the 2-position can be intuitively understood due to the steric hindrance by the substituent at the 2-position.

**Figure 9:**
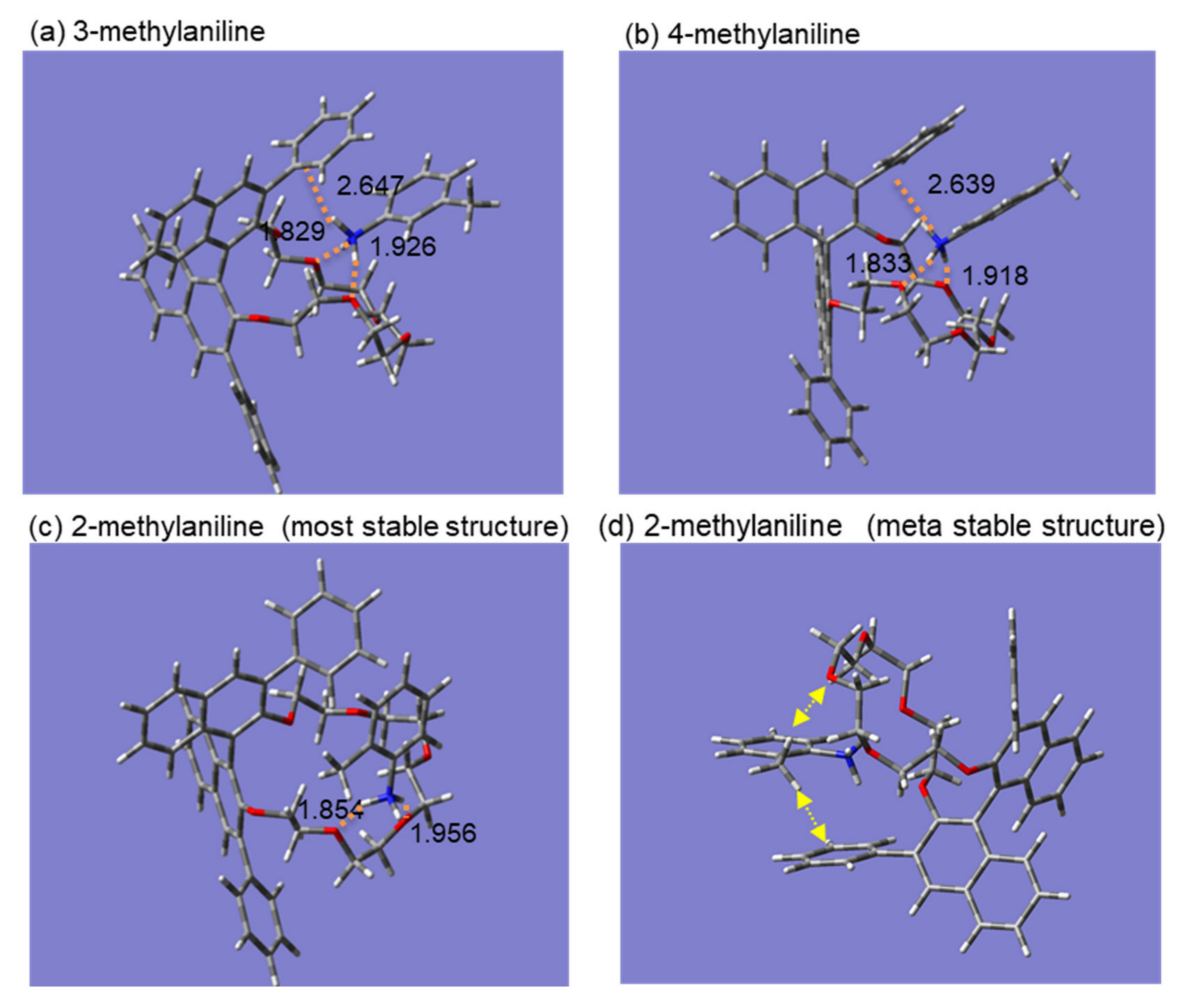
Calculated interaction models of methylanilinium-ion-substituted isomers and **22**. (**a**) 3-methylaniline, (**b**) 4-methylaniline, (**c**,**d**) 2-methylaniline.

To gain an insight into the assumed steric hindrance, the hydrogen-bonding pattern was further simulated. We predicted 3- and 4-methylaniline to make two hydrogen bonds with oxygen atoms at positions 6 and 15 of **22** (Figure 9a,b), and 2-methylaniline to make bonds at positions 6 and 9 (Figure 9c) as the global minimum (the most stable) structure. To see what would be expected if 2-methylaniline would make hydrogen bonds with oxygen atoms at positions 6 and 15, the calculation was performed starting from the above hydrogen bonding form as an initial aggregate structure. While a local minimum keeping the two hydrogen bonds was predicted, the stabilization energy was less than that of the global minimum, (Figure 9d) by 1.78 kcal/mol presumably due to the crowded structure, in particular of the surroundings of the methyl group (yellow arrows). Thus, it seems reasonable that 2-methylaniline is forced to make two hydrogen bonds with oxygen atoms numbered 6 and 9.

### 2.3. Insight into the Interaction between CR (+) Ligand and Aniline by Quantum Chemical Calculation

**Scheme 6:**
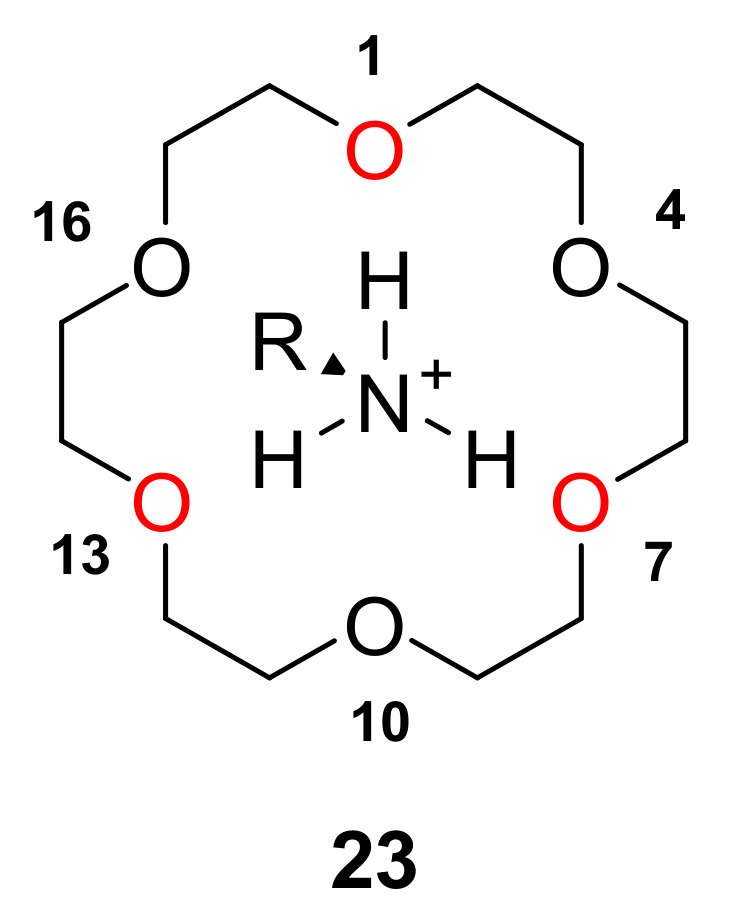
Interaction form between 1, 4, 7, 10, 13, 16-hexaoxycyclooctadecane (18-Crown-6) and ammonium ions.

The stable aggregate structures between the CR-I(+) ligand (**22**) and the substituted anilines were predicted by quantum chemical calculation as mentioned in Section 2.2. The calculation suggested that the 3- and 4-substituted anilines make two hydrogen-bonds with the oxygen atoms at the 9 and 12 positions, respectively, of the crown ether. This interaction model is different from the three-point binding model as described in Scheme 6. In this section, we discuss how the interaction form varies depending on the crown ether and the ammonium structures by in silico experiments.

#### 2.3.1. The Calculation Study of Various Crown Ethers and Anilines

**Figure 10:**
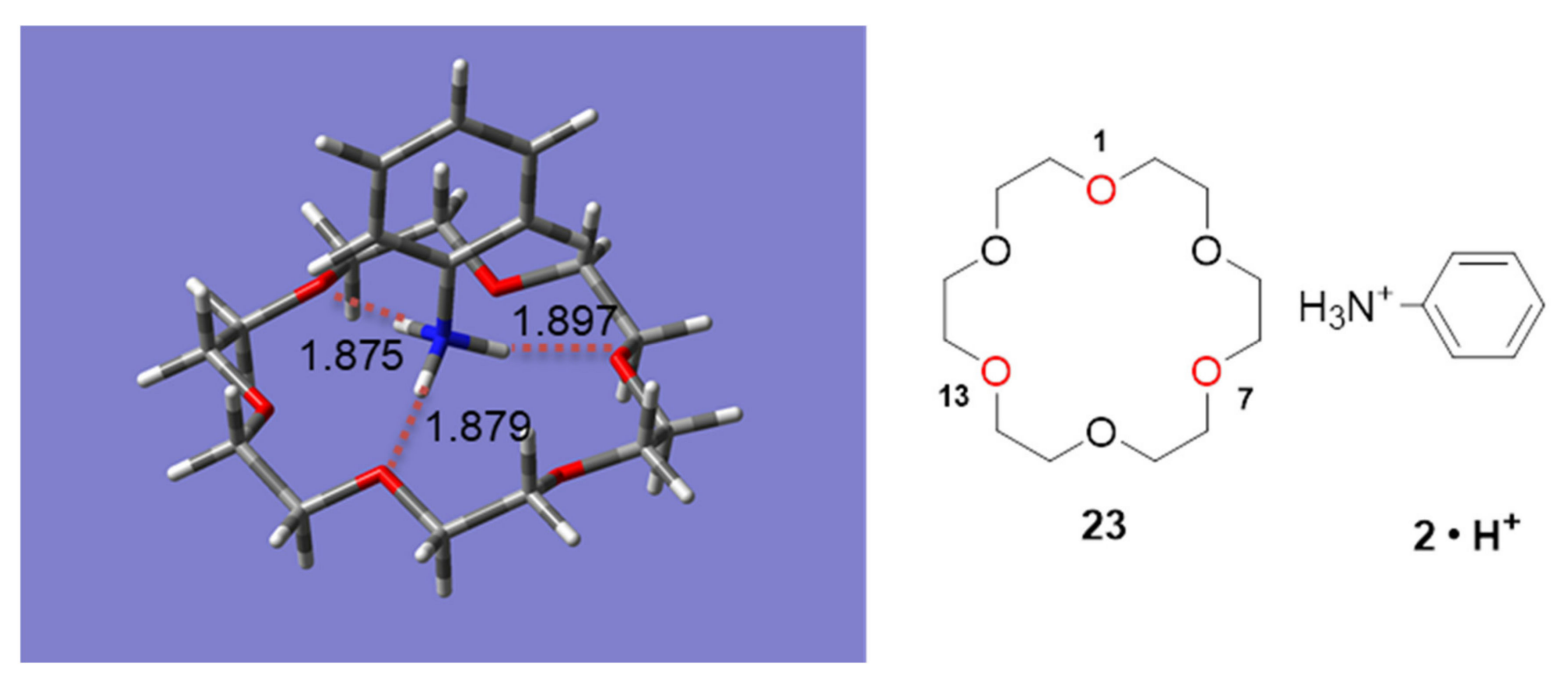
The structures of **23** and **2**·H^+^ (**right**) and the calculated stable aggregate between them (**left**). The number in the figure indicates the interatomic distance (angstrom).

To clarify whether this discrepancy (two hydrogen bonding vs. three hydrogen bonding) is due to the calculation method or the structure of the CR-I ligand having a symmetricity different from that of 18-crown-6, we performed calculations for several kinds of crown ethers with aniline. First, the most stable aggregated structure between 18-crown-6 (**23**) and aniline is shown in Figure 10. This apparently shows three-point binding with alternating oxygen atoms of **23** and **2**·H^+^. It seems likely from the result that the two-point binding model is not due to our calculation method but to the structure of **22**.

**Figure 11:**
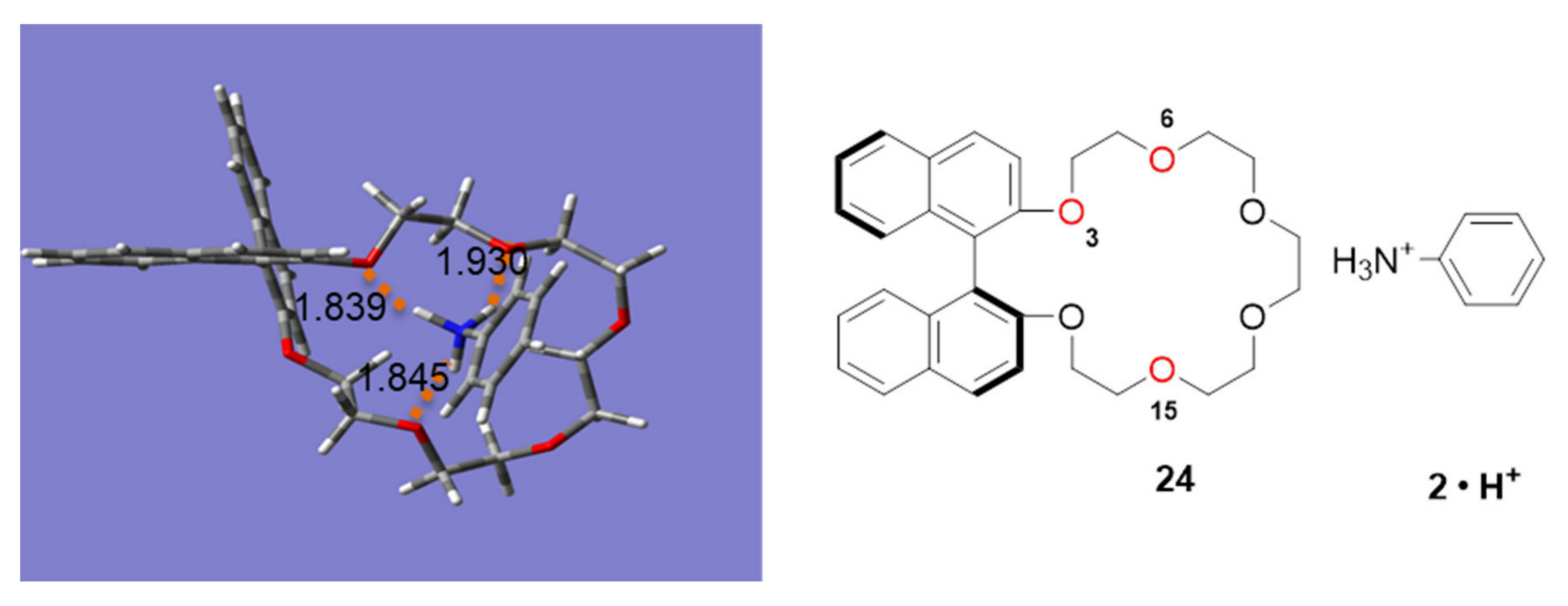
The structures of **24** and **2**·H^+^ (**right**) and calculated stable aggregate between them (**left**).

Next, the aggregate between the crown ether incorporating a 1, 1′-binaphthyl moiety without phenyl substituents at 3, 3′-positions (**24**), and ****2**·H^+^** was performed. In the resulting model, all three protons on the protonated nitrogen make hydrogen bonds with three of the crown ether oxygen atoms. However, unlike the case of **23** (Figure 10), the hydrogen bonds were predicted to be with oxygen atoms at the 3-, 6-, and 15-positions (Figure 11). This crown ether is no longer an 18-crown-6 (**23**) but a 20-crown-6 (**24**) by incorporating a 1, 1′-binaththyl moiety and has *C_2_* symmetry instead of the *C_3_* symmetry of **23**.

**Figure 12:**
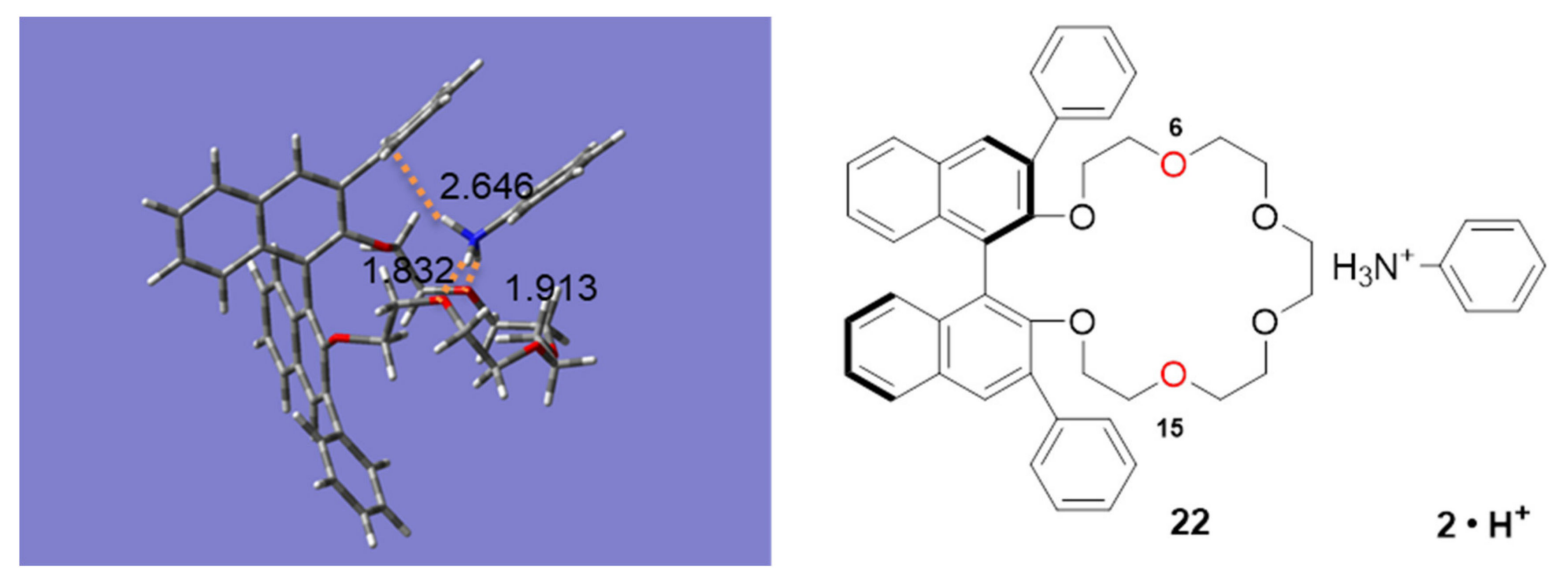
The structures of **22** and **2**·H^+^ (**right**) and calculated stable aggregate between them (**left**).

The novel pattern of ammonium-crown ether aggregation is likely the result of the crown ether structure. We presumed that, in the case of **22**, the hydrogen bonding with the oxygen atom at position 3 of **22** in the aggregate does no more work owing to the steric congestion caused by the phenyl groups at the 3- and 3′-positions resulting in a two-point binding interaction with two oxygen atoms at the 6 and 15 positions as shown in Figure 12. The remaining one hydrogen appears to form an NH-π bond as an alternative interaction.

Because the anilines substituted at position 2 further increase the steric hindrance, the crown ether oxygens accepting hydrogen bonds move further apart from the binaphthyl moiety, i.e., the oxygen atoms at positions 6 and 9 (Figure 9c); the third proton has no counterpart of hydrogen bonding; and the stabilization energy is smaller than for the 3- and 4-substituted anilines. The above is our understanding of the interaction pattern varying according to the structural change of both the crown ether and analytes.

#### 2.3.2. Interaction of Chiral Amino Acids and CR-I(+)

**Figure 13:**
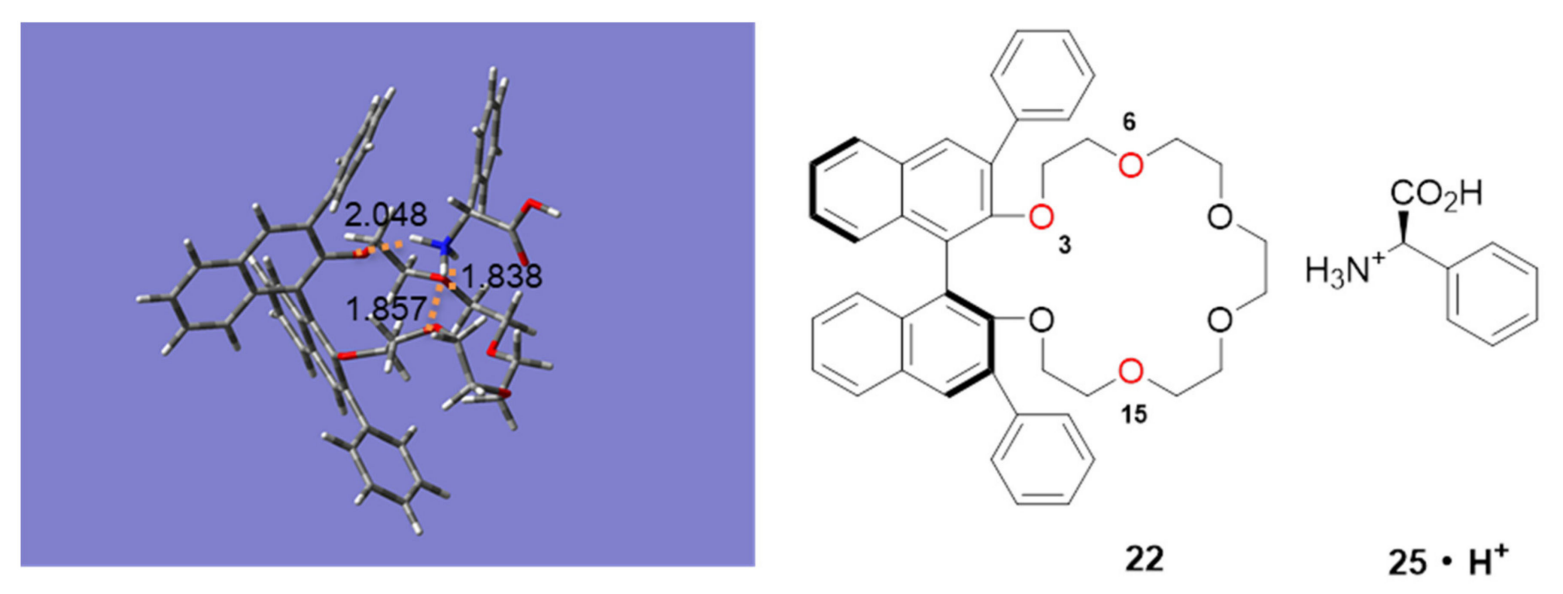
The structures of **22** and **25**·H^+^ (**right**) and calculated stable aggregate between them (**left**).

**Figure 14:**
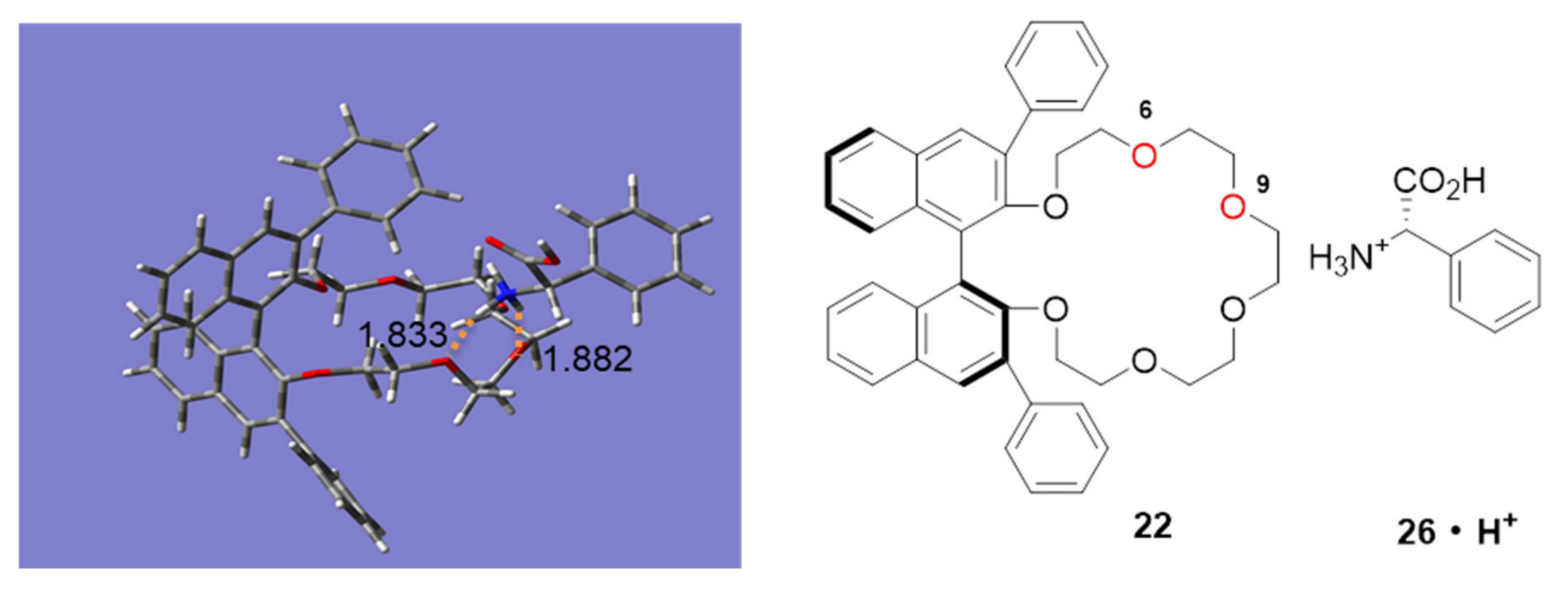
The structures of **22** and **26**·H^+^ (**right**) and calculated stable aggregate between them (**left**).

While three-point binding mechanisms based on Cram’s model have been usually supposed to rationalize chiral recognition, this study predicted an anomalous two-point binding mechanism based on the quantum chemical calculations for the aggregate model. To confirm whether the similar two-point binding model was also predicted in chiral recognition, the calculation for the aggregate between (*R*) or (*S*)-phenylglycine (**25**, **26**) and **22** was performed. The stabilization energies and interaction models of each enantiomer and 22 are shown in Table 6, Figure 13 and Figure 14. There was a good agreement between the stabilization energy of the aggregate and the elution sequence obtained from chromatography—that is, the stabilization energy of the more strongly retained (*S*)-enantiomer with **22** was calculated to be −23.91 kcal/mol, and the weakly retained (*R*)-enantiomer was calculated to be −19.41 kcal/mol.

The aggregate model of (*R*)-enantiomer **25** and **22** is similar to Figure 13 with an anomalous three-point binding mechanism in which three hydrogens are bonding with three oxygens at the 3, 6, and inner 15 positions in the crown ether ring. The aggregate model of (*S*)-enantiomer **26** and **22** (Figure 14) is rather similar to the 2-substituted aniline model shown in Figure 9c. The two hydrogen bonds with two oxygens at the 6 and 9 positions, which were predicted for the aggregate of 2-substituted anilines, are thought to be due to an unconfirmed effect, for example, the influence of the polar carboxyl group of amino acids.

## 3. Materials and Methods

### 3.1. Apparatus

Chromatography was performed using an HPLC consisting of an LC 20AD Liquid Chromatograph, SOL 20AC Auto Sampler, DGU-20A3R Degassing Unit, SPD-M20A Diode Array Detector, CTO-20AC Column Oven, and CBM-20A Communications Bus Module, all purchased from Shimadzu Corporation (Kyoto, Japan), and the data were processed with LCsolution, Shimadzu Corporation. The operating flow rate, UV wavelength, and temperature are described in each table. The sample material was dissolved into the mobile phase at a ca. 1 mg/mL concentration, and 1 μL or 2 μL of the sample solution was injected. CROWNPAK CR-I (-) (150 mm × 3 mm I.D., 5 μm packing) was used as a brand new column from Daicel Corporation (Tokyo, Japan).

### 3.2. Chemicals

Aniline and 60% perchloric acid were purchased from Nacalai Tesque (Kyoto, Japan); 2-aminophenol and 4-aminophenol, from Wako Pure Chemical (Osaka, Japan); and all other substituted anilines, benzyl amine, and methylbenzylamines, from Tokyo Chemical Industry Co., Ltd. (Tokyo, Japan) Liquid chromatography (LC) grade ACN “Acetonitrile-plus-” was purchased from Kanto Chemical Co., Inc. (Tokyo, Japan) Pure water was prepared with Millipore Elix Essential 3 UV (Burlington, USA).

### 3.3. Eluent

The water/ACN mixtures were prepared based on the weight. For example, the 70:30 (*v/v*) mixture was prepared by mixing 234.9 g of ACN and 700.0 g of water. 20 mM perchloric acid in the above mixture was prepared as follows. Perchloric acid (60%, titrated to confirm the concentration) was weighed targeting 1674.3 mg. It was transferred into a 500 mL volumetric flask with the above ACN/water mixture and around 225 µL of ACN to compensate for the water contained in the perchloric acid up to the graduation marking.

### 3.4. HPLC Conditions

#### 3.4.1. Analysis on a CROWNPAK CR-I (-) Column

The analysis on a CROWNPAK CR-I was performed under the following conditions (except Figure 4):

Column: CROWNPAK CR-I (-) (Daicel Corporation) Size: 150 × 3 mm ID

Eluent A: 20 mM HClO_4_ in 70:30 (*v/v*) mixture of H_2_O and ACN

Eluent B: 20 mM HClO_4_ in 10:90 *(v/v)* mixture of H_2_O and ACN

Eluent C: 100/100/4/1 *(v/v/v/v)* mixture of n-hexane, ethanol, water, and trifluoroacetic acid

Flow rate: 0.25 mL/min., Temperature: 30 °C, and Detection: UV 254 nm under all conditions.

V0 was determined with formamide as the marker to be 0.809 mL.

#### 3.4.2. Analysis of ODS Column

Column: L-Column A5206 290-W, Size: 250 mm × 4.6 mm ID (Chemicals Evaluation Research Institution, Tokyo, Japan)

Eluent: 20 mM phosphoric acid, 30 mM triethylamine in water/ACN 7:3(*v/v*)

Flow rate: 1.0 mL/min., Temperature: 30 °C, and Detection: UV 254 nm

V_0_ was determined with foramide as the marker to be 2.698 mL.

### 3.5. Calculation

Molecular orbital calculations were performed with the Gaussian16 programs [20]. The geometries of all the compounds were optimized with the density functional theory (DFT) method with the hybrid Becke3 parameter hybrid (B3) [21] as a functional and 1991 [22] by Perdew and Wang (PW91) as a gradient correction functional by using the 6-31G (d) [23] basis (denoted as B3PW91/6-31G(d)). The solvent effect for each molecule used a polarized continuum model (PCM) [24]. The dielectric constant was *ε* = 78.39, assuming that the solvent was water. Theoretical harmonic vibrational frequencies were obtained from analytical second derivatives calculated at the B3PW91/6-31G(d) level and performed to confirm that the structure was stable.

## 4. Conclusions

The achiral selectivity of CROWNPAK CR-I, an immobilized crown ether type chiral stationary phase for isomeric substituted anilines, and the related analytes was studied. Fluids of a wide polarity range can be adopted as the eluent, which made it possible to control the retention of the analytes with different polarities. While this exhibited a good recognition of positional isomeric anilines, there was a remarkably smaller retention of 2-substituted aniline compared to other isomers as the outstanding feature of the phase. For the purpose of understanding the interaction occurring here, we attempted to predict the structure of the aggregate between the crown ether and methyl- or chloro-substituted aniline through quantum chemical calculations.

The results suggested that the protonated anilines make two hydrogen bonds with two of the crown ether oxygen atoms in two patterns (two-point binding mechanisms); aniline, 3-substituted anilines, and 4-substituted anilines with oxygen atoms at No.6 and No.15; and 2-substituted anilines with oxygen atoms at No.6 and No.9. This is different from the three-point binding mechanism that is well accepted for 18-Crown-6, and further simulation study suggested that a crown-6 with incorporated 1,1′-binaphthyl moiety takes an anomalous three-points binding mechanism. When two phenyl groups are substituted at the 3 and 3′ positions in the naphthalene ring, it takes a two-point binding model. Finally, quantum chemical calculations were applied to a chiral amino acid and predicted the correct enantiomeric elution order, and two types of hydrogen bonding models were predicted.

## Figures and Tables

**Table 1:** Retention times and retention factors of aniline (**2**) and substituted anilines (**3**–**8**) on a CROWNPAK CR-I(-) column under eluent conditions with the three types of eluent ^1^.

Analytes	Retention Time (Retention Factor ^2^)		
	Eluent (A) ^3^	Eluent (B) ^3^	Eluent (C) ^3^
aniline (**2**)	7.55 min (1.33)	10.52 min (2.25)	7.80 min (1.41)
2-methylaniline (**3**)	5.19 min (0.60)	4.74 min (0.47)	4.44 min (0.37)
3-methylaniline (**4**)	10.95 min (2.38)	11.23 min (2.47)	7.18 min (1.22)
4-methylaniline (**5**)	12.46 min (2.85)	12.68 min (2.92)	8.41 min (1.60)
2-chloroaniline (**6**)	8.12 min (1.51)	4.44 min (0.37)	4.38 min (0.36)
3-chloroaniline (**7**)	12.89 min (2.98)	12.14 min (2.75)	6.08 min (0.88)
4-chloroaniline (**8**)	14.57 min (3.50)	15.27 min (3.72)	7.62 min (1.35)

^1^ Chromatograms are shown in Figure 1, and the structures of the analytes are shown in Scheme 2. ^2^ retention factors are described in parentheses. ^3^ The eluent compositions are described in the text.

**Table 2:** Retention times and retention factors of aniline (**2**) and substituted anilines (**9**–**17**) on a CROWNPAK CR-I(-) column with three types of eluent ^1^.

Analytes	Retention Time (Retention Factor ^2^)		
	Eluent (A) ^3^	Eluent (B) ^3^	Eluent (C) ^3^
aniline (**2**)	7.55 min (1.33)	10.52 min (2.25)	7.80 min (1.41)
2-nitroaniline (**9**)	15.00 min (3.63)	3.78 min (0.17)	5.24 min (0.62)
3-nitroaniline (**10**)	11.88 min (2.67)	8.89 min (1.75)	6.24 min (0.93)
4-nitroaniline (**11**)	13.53 min (3.18)	4.04 min (0.25)	6.62 min (1.05)
2-hydroxyaniline (**12**)	6.19 min (0.91)	7.96 min (1.46)	8.72 min (1.70)
3-hydroxyaniline (**13**)	7.55 min (1.33)	13.02 min (3.02)	12.14 min (2.75)
4-hydroxyaniline (**14**)	7.41 min (1.29)	13.13 min (3.06)	12.42 min (2.84)
2-carboxyaniline (**15**)	5.85 min (0.81)	3.50 min (0.08)	5.12 min (0.58)
3-carboxyaniline (**16**)	7.49 min (1.31)	13.18 min (3.07)	9.21 min (1.85)
4-carboxyaniline (**17**)	6.90 min (1.13)	10.40 min (2.21)	6.51 min (1.01)

^1^ The structures of the analytes are shown in Scheme 3. ^2^ Retention factors are described in parentheses. ^3^ The eluent compositions are given in the text.

**Table 3:** Retention times and retention factors of aniline (**2**) and substituted anilines (**3**–**8**) and (**12**–**14**) on an octadecylsilyl (ODS) column ^1^.

Analytes	Retention Time (Retention Factor ^2^) ^3^
aniline (**2**)	7.99 min (1.96)
2-methylaniline (**3**)	12.57 min (3.66)
3-methylaniline (**4**)	12.86 min (3.77)
4-methylaniline (**5**)	12.26 min (3.55)
2-chloroaniline (**6**)	22.82 min (7.46)
3-chloroaniline (**7**)	21.37 min (6.92)
4-chloroaniline (**8**)	18.81 min (5.97)
2-hydroxyaniline (**12**)	4.56 min (0.69)
3-hydroxyaniline (**13**)	3.81 min (0.41)
4-hydroxyaniline (**14**)	3.23 min (0.20)

^1^ The structures of analytes are shown in Scheme 2 and Scheme 3. ^2^ Retention factors are described in parentheses. ^3^ The analytical conditions are given in Section 3. Materials and Methods.

**Table 4:** Retention times and retention factors of benzylamine (**18**) and methyl-substituted benzylamines (**19**–**21**) on a CROWNPAK CR-I(-) column with the three types of eluent ^1^.

Analytes	Retention Time (Retention Factor ^2^)		
	Eluent (A) ^3^	Eluent (B) ^3^	Eluent (C) ^3^
benzylamine (**18**)	9.46 min (1.92)	11.32 min (2.50)	10.71 min (2.31)
2-methylbenzylamine (**19**)	13.33 min (3.12)	11.71 min (2.62)	10.75 min (2.32)
3-methylbenzylamine (**20**)	15.65 min (3.84)	13.11 min (3.05)	10.11 min (2.12)
4-methylbenzylamine (**21**)	13.52 min (3.18)	11.25 min (2.48)	10.04 min (2.10)

^1^ The structures of the analytes are shown in Scheme 4. ^2^ Retention factors are described in parentheses. ^3^ The eluent compositions are described in the text.

**Table 5:** Stabilization energy of aniline, methylanilines, and chloroanilines.

Analytes	Stabilization Energy, ΔE (kcal/mol)
aniline (**2**)	25.31
2-methylaniline (**3**)	24.51
3-methylaniline (**4**)	24.98
4-methylaniline (**5**)	24.85
2-chloroaniline (**6**)	37.48
3-chloroaniline (**7**)	47.04
4-chloroaniline (**8**)	48.70

**Table 6:** The stabilization energies of phenylglycine enantiomers and chiral crown ether **22**.

Chiral. Crown Ether	Amino Acid	Stabilization Energy, ΔE (kcal/mol)	Elution Order ^1^
**22**	*(R)*-Phenylglycine (**25**)	19.41	1st
**22**	*(S)*-Phenylglycine (**26**)	23.91	2nd

^1^ The chromatographic condition is described in [19].

## Data Availability

Data sharing, not applicable.

## Sample Availability

Samples of the compounds are not available from the authors.
